# Draft Genome Sequences of Two Pairs of Human Intestinal *Bifidobacterium longum* subsp. *longum* Strains, 44B and 1-6B and 35B and 2-2B, Consecutively Isolated from Two Children after a 5-Year Time Period

**DOI:** 10.1128/genomeA.00234-13

**Published:** 2013-05-16

**Authors:** A. N. Shkoporov, B. A. Efimov, E. V. Khokhlova, A. V. Chaplin, L. I. Kafarskaya, A. S. Durkin, J. McCorrison, M. Torralba, M. Gillis, G. Sutton, D. B. Weibel, K. E. Nelson, V. V. Smeianov

**Affiliations:** Department of Microbiology and Virology, The Russian National Research Medical University, Moscow, Russian Federationa;; The J. Craig Venter Institute, Rockville, Maryland, USAb;; Department of Biochemistry, University of Wisconsin-Madison, Madison, Wisconsin, USAc

## Abstract

We report the genome sequences of four isolates of a human gut symbiont, *Bifidobacterium longum*. Strains 44B and 35B were isolated from two 1-year-old infants, while 1-6B and 2-2B were isolated from the same children 5 years later. The sequences permit investigations of factors enabling long-term colonization of bifidobacteria.

## GENOME ANNOUNCEMENT

Members of the genus *Bifidobacterium* are nonmotile, non-spore-forming, mostly catalase-negative, Gram-positive polymorphic anaerobic rods, which metabolize glucose via the fructose-6-phosphate shunt (1). In human newborns bifidobacteria are among the pioneering microbial colonizers that dominate the intestinal microbiota during breastfeeding (2). They also constitute a significant portion of the microbial community in adults (3). Bifidobacteria are believed to contribute to the host well-being and are being increasingly used as probiotics (4). Yet, understanding of colonization factors of bifidobacteria remains limited (5). Earlier studies suggested that bifidobacteria inhabiting the infant intestine are replaced and/or supplemented by other strains during the transition to adulthood (6–8). This succession is believed to be caused by the change from a milk-based diet to solid foods (9). However, we previously found that some children at 6 years of age were colonized by strains of *Bifidobacterium longum* subsp. *longum* that were indistinguishable from strains isolated 5 years earlier (10).

To identify genetic features responsible for the temporal ecological stability of the strains, we sequenced the genomes of two presumptive ancestor-descendant pairs of *B. longum* subsp. *longum* strains: 44B and 1-6B (both from child 1), and 35B and 2-2B (both from child 2), isolated at the ages of 1 and 6 years, respectively. The sequencing was performed on a 454 FLX system at the J. Craig Venter Institute (JCVI). The assemblies were generated using either Newbler 2.6 or CA 7.0 assemblers. The annotation was performed using the JCVI prokaryotic annotation pipeline. The generated genomes were compared using progressive Mauve (11).

The preliminary comparison identified several genomic islands present in all four strains but not found simultaneously in any of the currently available bifidobacterial genomes. These islands include potential colonization and survival factors, such as adhesins genes, exopolysaccharide (EPS) biosynthesis clusters, polysaccharide utilization genes, and toxin-antitoxin modules. Another intriguing observation is the presence of highly similar type I CRISPR-Cas modules (12) in the strains isolated from the different children. The architecture of the modules seems to be unique for these strains. The CRISPR-Cas system provides for an adaptive defense against bacteriophages and plasmids that is guided by previously acquired short spacers that direct the cleavage of invading nucleic acids. The spacers in strains 44B, 1-6B and 2-2B match to a number of plasmid sequences, such as p4M of *Bifidobacterium pseudocatenulatum* (13).

Phylogenetic analysis indicated the genomes within the pairs are closely related, and while strains 35B and 2-2B possibly constitute a true ancestor-descendant pair, strains 44B and 1-6B appear to have evolved independently from a common ancestor. This observation suggests that at the time of the first sampling at least two very close lineages of bifidobacteria coexisted in the intestine of child 1.

To summarize, this is the first report of high-quality draft genome sequences of intestinal symbionts isolated from the same human individuals repeatedly sampled after a significant time period. These data provide insight into the *in vivo* evolution of bifidobacteria and also may permit identification of genomic features specific to the long-term colonizers.

### Nucleotide sequence accession numbers.

The sequences described were submitted to GenBank under the accession numbers given in Table 1.

**TABLE 1:** Genome features of the sequenced strains

Strain	Genome size (Mb)	Contigs	Genome coverage	Proteins	Accession no.
*B. longum* subsp. *longum* 44B	2.56	62	25×	2,262	AJTM00000000
*B. longum* subsp. *longum* 1-6B	2.69	171	14×	2,425	AJTF00000000
*B. longum* subsp. *longum* 35B	2.51	131	11×	2,260	AJTI00000000
*B. longum* subsp. *longum* 2-2B	2.63	141	11×	2,412	AJTJ00000000
